# Successful Use of Recombinant Tissue Plasminogen Activator in an Extremely Low Birth Weight Premature Infant for the Resolution of Multiple Thrombi

**DOI:** 10.1055/a-2780-3344

**Published:** 2026-01-14

**Authors:** Kartik Mody, Christine Wade, Becky Micetic, Myleanna Vernon, Natalie Wade

**Affiliations:** 1 Department of Neonatology, Pediatrix/Arizona Neonatology, Phoenix, Arizona, United States; 2 Neonatal Intensive Care Unit, Banner – University Medical Center Phoenix, Phoenix, Arizona, United States; 3 College of Medicine, University of Arizona College of Medicine, Phoenix, Arizona, United States; 4 Department of Pediatrics, Creighton University School of Medicine, Phoenix, Arizona, United States

**Keywords:** infant, extremely premature, thrombosis, thrombolytic therapy, heparin, recombinant tissue plasminogen activator, heparin, low-molecular-weight

## Abstract

**Introduction:**

The literature is limited regarding the use of recombinant tissue plasminogen activator (r-tPA) in premature infants. We describe the use of r-tPA to treat life-threatening intra- and extracardiac thrombi in a very low birth weight patient born at 23 weeks of gestational age.

**Case Report:**

An extremely premature infant was diagnosed with multiple thrombi at 4 weeks of age. The acute phase of treatment was managed with an infusion of r-tPA followed by unfractionated heparin, then low-molecular-weight heparin for continued anticoagulation. The patient did not experience any side effects associated with the therapies and was discharged home.

**Conclusion:**

Thrombotic events in neonates, though rare, are being increasingly identified due to improved survival of premature infants, enhanced diagnostic modalities, and the widespread use of central venous catheters. Heparin remains the standard of care in neonates for anticoagulation. Thrombolytic therapy with agents such as r-tPA, though less frequently employed, may be lifesaving in certain instances, such as the one presented in this case report.

## Background

The occurrence of thrombosis in a newborn is infrequent and usually is a complication of a primary illness or prematurity. Extremely premature infants have immature coagulation systems and commonly require central line catheterization; the most common risk factor for clot formation. 1 2 3 4 5 Additionally, technological advances have led to an increased detection of thrombi. 6 7 Literature from the 1990s and early 2000s reported the incidence of neonatal thrombosis as 2.4 per 1,000 neonatal intensive care unit (NICU) admissions, while newer registry data published between 2018 and 2020 demonstrate a significant increase, with incidence ranging from 6.9 to 15 per 1,000. 6 7 8 9 10 11

Anticoagulation with unfractionated heparin (UFH) and low-molecular-weight heparin (LMWH) is standard in the treatment of infants and children with thrombosis. However, the dosage and optimal duration of therapy with the use of recombinant tissue plasminogen activator (r-tPA) as a thrombolytic agent are not well-studied in children. 12 13 There is a well-documented increased risk of intraventricular hemorrhage (IVH) and other sequelae for thrombolysis treatment in infants, creating uncertainty and hesitancy surrounding treatment options. 14

## Case Report

**Video 1** Echocardiogram day of life 29. Footage of a large linear homogenous thrombus located in the right ventricular outflow tract extending into the proximal right pulmonary artery. The mass measures 0.5 cm by 3 cm.


A woman presented with preterm premature rupture of membranes in the setting of cervical insufficiency with cerclage placement at our Southwestern regional medical center, with over 7,000 deliveries per year. Following a week of ruptured membranes, an emergent cesarean section was performed for fetal distress. An extremely premature male infant was delivered at 23 ^2/7^ weeks, weighing 600 grams. In the days after birth, the placental examination showed marked acute chorioamnionitis and funisitis.

The infant was intubated at birth and placed on high-frequency jet ventilation after the administration of surfactant. Umbilical venous and arterial catheters were inserted upon admission to the NICU. After placement, the tip of the umbilical arterial catheter (UAC) was visualized at the T8 vertebral level, and the umbilical venous catheter (UVC) was seemingly positioned in the portal vein. Due to the extreme prematurity and the inability to secure other vascular access, the UVC was retracted and utilized as a low-lying catheter. On day of life (DOL) 1, total parenteral nutrition containing heparin was infused through the UVC, and 1/2 sodium acetate with heparin through the UAC per unit protocol.

After two previous unsuccessful attempts, peripherally inserted central catheter (PICC) placement occurred on DOL 8. The NICU PICC team placed a 1 French catheter in the right lower extremity, with placement verified by X-ray in the inferior vena cava. Both the UAC and UVC were then discontinued at this time. Enteral feedings were started on DOL 3 and advanced slowly due to intermittent feeding intolerance. On DOL 13, total parenteral nutrition was replaced with half sodium acetate containing heparin to keep the PICC patent. There was clinical suspicion of a symptomatic patent ductus arteriosus, and an echocardiogram (ECHO) was performed on DOL 13, which showed a small fenestrated atrial septum with bidirectional shunting and a right ventricular pressure of less than half of systemic. No evidence of thrombus, clots, or a patent ductus arteriosus was visualized at this time. Feeding intolerance and the need for sedation with high-frequency ventilation necessitated the use of the PICC until DOL 23.

**Fig. 1:**
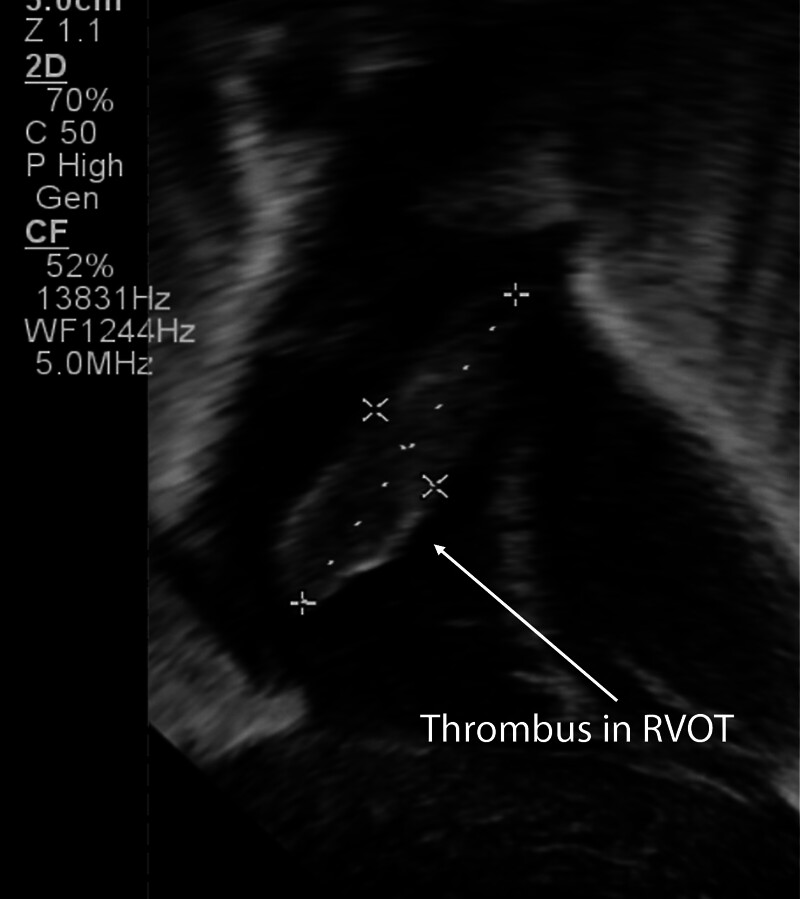
Echocardiogram day of life 29. Initial cardiac thrombus identified in the right ventricular outflow tract (RVOT).

On DOL 29, at a corrected gestational age of 27 ^3/7^ weeks, increased oxygen needs were present despite changes to the high-frequency jet ventilation. Dopamine, inhaled nitric oxide, and hydrocortisone were started for clinical persistent pulmonary hypertension and systemic hypotension. A peripheral arterial line was placed, and 0.45 normal saline with heparin was infused through it for patency as per NICU protocol; prophylactic oxacillin and gentamicin were initiated for suspected sepsis, and feedings were discontinued. A head ultrasound (US) was performed without evidence of intracranial hemorrhage appreciated. An ECHO revealed a single large linear thrombus extending from the right ventricular outflow tract into the proximal right pulmonary artery, measuring 0.5 cm × 3 cm ( Fig. 1 ). Pediatric cardiology and hematology were consulted regarding options for intervention. On DOL 30, additional scans revealed the presence of a new small clot in the heart as well as an extracardiac thrombus in the mid to distal aorta extending to the right iliac artery measuring 3.5 cm. The progression of imaging results, lab values, and medications during DOL 29 to 33 can be found in Table 1 . video 1

**Table 1:** Five-day overview of the monitoring and treatment regimen for the thrombi

Day of life	Imaging results	Laboratory values	Medications and transfusions
29	**ECHO** • Mass ▪ Description: Large, linear, homogenous ▪ Size: 0.5 cm × 3 cm ▪ Location: From the RVOT into the proximal RPA with minimal antegrade flow into the RPA and forward flow into the LPA **Head US** • No hemorrhage or ventriculomegaly, appearance consistent with prematurity	• Platelets 112–130 × 10 ^9^ /L • PT 13.2 seconds • APTT 43.6 seconds • Fibrinogen 240 mg/dL	• Transfused PRBC (goal Hct >30%) • UFH infusion started at 28 U/kg/h
30	**ECHO** • Mass #1 ▪ Description: Large, linear homogenous, mobile ▪ Size: 0.4 cm × 2 cm ▪ Location: From the RVOT into the proximal RPA with improved antegrade flow into the RPA and forward flow in the LPA • Mass #2 ▪ Description: Small echogenic ▪ Size: 0.2 cm × 0.3 cm ▪ Location: Near the IVC–RA junction **Abdominal US** • Long-segment non-occlusive thrombus involving the mid to distal aorta with propagation into the right iliac artery *6 hours after r-tPA began* **ECHO** • Mass #1 ▪ Description: Large saddle mass ▪ Location: In the distal main PA extending into the proximal branch pulmonary arteries, no RVOT obstruction or thrombus • Mass #2 ▪ Description: Small, non-obstructive echogenic ▪ Location: Near IVC–RA junction, with filamentous strands into the RA **Aorta US** • Echogenic focus in the distal aorta extending into right and left common iliac arteries with maintained Doppler signal in the vessels	• Platelets 53 × 10 ^9^ /L • Anti-Xa 0.16 IU/mL • Antithrombin III 24% • Fibrinogen 217 mg/dL *4 hours after r-tPA began* • Platelets 98 × 10 ^9^ /L (goal >100 × 10 ^9^ /L) • PT 13.5 seconds • APTT 107.9 seconds • Fibrinogen 176 mg/dL (goal >100 mg/dL)	• Transfused PRBC, platelets, plasma ×2 • tPA infusion at 0.06 mg/kg/h × 6 hours • UFH infusion reduced to 10 U/kg/h
31	**ECHO** • Mass #1 ▪ Description: Echodense ▪ Size: 0.3 cm × 0.46 cm ▪ Location: Distal main PA extending into proximal RPA, no RVOT obstruction or thrombus • No obvious density appreciated in distal IVC/RA **Aorta US** • Echogenic thrombus in mid to distal aorta extending inferiorly to right common iliac artery, slightly less prominent on the prior exam	• Platelets 75–206 × 10 ^9^ /L • PT 12.0–13.7 seconds • APTT 56.9–25.3 seconds • Fibrinogen 148–199 mg/dL	• Transfused PRBC ×2, platelets ×2, plasma • r-tPA infusion at 0.06 mg/kg/h × 6 hours
32	**ECHO** • Previously noted large thrombus in RVOT is not seen, a thin layer of echogenicity is visualized in the proximal RPA with no flow obstruction • No obvious residual IVC/RA echodensity **Aorta US** • Stable non-occlusive thrombus within the mid to distal aorta extending inferiorly into the right common iliac artery **Head US** • No evidence of intracranial hemorrhage	• Platelets and fibrinogen within hematology recommendations • PT 12.3–13.6 seconds • APTT 30.8–84.5 seconds	• tPA therapy stopped, and UFH increased to a therapeutic level of 30 U/kg/h
33	**Aorta US** • Echogenic thrombus again seen in the mid to distal aorta extending inferiorly to the right common iliac artery	• Platelets and fibrinogen within hematology recommendation • Anti-Xa 0.22 IU/mL (goal 0.3–0.7 IU/mL)	• Transfused PRBC

Abbreviations: APTT, activated partial thromboplastin time; Hct, hematocrit; IVC, inferior vena cava; LPA, left pulmonary artery; PA, pulmonary artery; PRBC, packed red blood cell; PT, prothrombin time; r-tPA, recombinant tissue plasminogen activator; RA, right atrium; RPA, right pulmonary artery; RVOT, right ventricular outflow tract, UFH, unfractionated heparin; US, ultrasound.

Initially, there was reluctance to utilize r-tPA for treatment related to the infant's small size (800 g), gestational age, and risk of life-threatening bleeding. Multiple discussions were held with the NICU medical team, specialists, and the family regarding the high mortality and morbidity associated with neonatal thrombosis, as well as the limited evidence available on the safety and efficacy in this population. Collaboratively, a decision was made to provide treatment with r-tPA. Rather than starting with a bolus dose, a continuous infusion was initiated over 6 hours at 0.06 mg/kg/h. Per hematology's recommendation, baseline levels of platelets and fibrinogen were drawn prior to r-tPA administration. After the infusions, they were monitored every 6 to 8 hours, and antithrombin III levels were obtained as needed.

A second round of r-tPA was infused on DOL 31, after imaging revealed a substantial decrease in the size of the thrombus in the pulmonary artery, and the second cardiac thrombus was no longer appreciated. On both DOL 32 and 33, an aortic US showed the non-occlusive thrombus in the mid-to-distal aorta, which had not significantly changed in appearance. A Doppler signal was seen throughout the aorta and the bilateral common iliac arteries. Resolution of the thrombus in the pulmonary artery was reported. Due to the resolution of the intracardiac thrombi, the multidisciplinary team decided to initiate long-term UFH therapy. Anti-Xa levels were monitored daily with a goal of achieving levels between 0.3 and 0.7 IU/mL. A new upper extremity PICC was placed for anticoagulation therapy.

Serial imaging continued to show a persistent, non-occlusive aortic thrombus extending into the right common iliac artery. However, over the course of nearly 16 weeks, this extracardiac thrombus decreased in size from 3.9 to 1 cm, as documented on the final scan at DOL 149. Progressive improvement in color Doppler visualization of flow throughout the aorta was also observed. There was no evidence of intracardiac clots noted on these follow-up scans. The UFH infusion was continued for a total duration of 7 weeks, followed by a transition to LMWH for an additional 5 weeks. On DOL 131, after almost 100 days of therapy, the heparin was discontinued, and 9 days later, the thrombophilia workup was within normal limits. A head US prior to discharge to screen for periventricular leukomalacia was normal. The infant was discharged home on DOL 156 at a corrected gestational age of 45 ^4/7^ weeks, weighing 4,995 g with continuous pulse oximetry and nasal cannula at 0.1 L of oxygen per minute. Two weeks after discharge, the infant had outpatient visits with hematology and pulmonology. The haematologist's exam was normal and repeat thrombophilia testing was recommended around 9 months of age. He had been successfully maintaining saturations of > 95% on 0.1 liter of oxygen per minute therefore, pulmonology did not wean support and recommended follow up in 2 months. An ECHO performed at 3 weeks post discharge demonstrated normal anatomy, absence of intracardiac clots, and resolving persistent pulmonary hypertension with right ventricular pressure < ½ systolic. A repeat ECHO was advised in 4 months to further monitor pulmonary artery pressure. At approximately 2 years of age, the mother reported normal development for his CGA, no further oxygen use, and complete resolution of the calcified extracardiac clot.

## Discussion

We describe the use of anticoagulation and thrombolytic treatment of intra- and extracardiac thrombi in a 4-week-old extremely low birth weight (ELBW) infant born at 23 weeks with a birth weight of 600 grams. Therapy included an r-tPA infusion in combination with UFH administration followed by LMWH. Central catheter-related thrombosis is a relatively common complication in ELBW infants, with incidence varying from 2% to 10% of patients with central venous catheters. 15 Endothelial damage to a vessel may initiate a thrombotic process, which can be further intensified when other risk factors are present. 1 16 17 These can include polycythemia, infection, dehydration, perinatal asphyxia, and prenatal inflammation associated with chorioamnionitis. 1 18 Our infant was at a high risk for thrombosis in view of preterm premature rupture of membranes, chorioamnionitis, and the presence of multiple central catheters. These factors may have contributed to the development of thrombi.

Historically, thrombolytic therapies have included streptokinase, urokinase, and r-tPA. 19 A literature review by Leong et al 20 showed blood clot resolution as high as 87.9% with the use of these agents in neonates. There is a lack of strong clinical evidence regarding the safety of r-tPA in premature infants, and the majority of the literature is confined to case reports. 12 13 15 20 21 22 23 24 25 26 In preterm neonates, treatment with r-tPA carries a substantial risk of bleeding. Consequently, its use is generally restricted to cases of severe, life-threatening thrombosis where the potential benefits outweigh the risks. 20 27 28 29

Although the pharmacokinetics for r-tPA have not been established in infants, the potential advantages include a short half-life, low antigenic properties, and increased plasminogen activation in the presence of fibrin. 19 30 31 32 The decreased level of plasminogen in neonates, which is approximately 50 to 75% less than in adults, slows the generation of plasmin and reduces the effect of thrombolytics. 13 33 34 Appropriate patient selection, including evaluation of the serious risks and potential benefits of a r-tPA infusion as well as the predicted outcome of the underlying disease, should be addressed individually.

Despite variations in neonatal r-tPA use, the approach mentioned in most studies involves a loading dose followed by continuous infusion. Similar to Kimura et al, we opted not to utilize a loading dose. 24 While some research suggests a conventional range of r-tPA at 0.1 to 0.6 mg/kg/h, others recommend low-dose therapy starting at 0.03 mg/kg/h with the option to titrate up to 0.06 mg/kg/h. 20 23 28 29 34 35 In view of extreme prematurity and risks of IVH, we chose to treat at the higher end of the low-dose regimen related to the infant's size and gestational age. Frequent monitoring of platelets, fibrinogen, and disseminated intravascular coagulation panels during attempted lysis of the clots was necessary. To increase success by providing adequate plasminogen, plasma was administered three times over the r-tPA infusion period (8–12 mL). 23 A head US was performed before r-tPA therapy to ensure eligibility and repeated after completion of the infusions as recommended in the literature. 14 25 36

Use of a thrombolytic agent like r-tPA alone does not prevent propagation. With the addition of heparin, the safe and rapid resolution of a clot can occur. 26 34 The novel medication regimen included an infusion of r-tPA at 0.06/mg/kg/h in combination with UFH at 10 U/kg/h. Literature has shown the efficacy of combination therapy when contraindications for use are adequately followed. 19 23 26 With a short half-life and studied safety, heparin is a frequently used anticoagulant in the pediatric population. 37 38 Utilizing UFH does require intravenous access and frequent blood samples to monitor therapeutic drug levels. 39 Additionally, there is a risk of drug-induced thrombocytopenia. 38 In our patient, UFH was started once the initial cardiac thrombus was identified and was reduced to 10 U/kg/h during the 2 days r-tPA was administered. After which, the dosage was increased to 30 U/kg/h to maintain the anticoagulated state and later changed to LMWH for maintenance. Anti-Xa levels were initially monitored every 8 hours and then daily with a target of 0.3 to 0.7 IU/mL while receiving continuing anticoagulant therapy. 40 41

## Conclusion

There are very few case studies that describe intra- and extracardiac thrombi in a 4-week-old ELBW infant born at 23 weeks. In this case, the benefits of clot resolution exceeded the risks of significant bleeding. Therefore, it was reasonable to consider r-tPA for thrombolysis in this setting. With the medication regimen that was administered, the thrombi were successfully lysed. Resolution of these clots using an unconventional dosing regimen, without experiencing the comorbidities of IVH or disseminated intravascular coagulation, was the unique aspect of this case.

## References

[1] Demirel N Aydin M Zenciroglu A Neonatal thrombo-embolism: Risk factors, clinical features and outcome Ann Trop Paediatr 2009 29 04 271 279 19941750 10.1179/027249309X12547917868961

[2] Tuckuviene R Christensen A L Helgestad J Johnsen S P Kristensen S R Pediatric venous and arterial noncerebral thromboembolism in Denmark: A nationwide population-based study J Pediatr 2011 159 04 663 669 21596390 10.1016/j.jpeds.2011.03.052

[3] Andrew M E Monagle P deVeber G Chan A K Thromboembolic disease and antithrombotic therapy in newborns Hematology (Am Soc Hematol Educ Program) 2001 358 374 11722993 10.1182/asheducation-2001.1.358

[4] Yang J Y Williams S Brandão L R Chan A K Neonatal and childhood right atrial thrombosis: Recognition and a risk-stratified treatment approach Blood Coagul Fibrinolysis 2010 21 04 301 307 20305543 10.1097/MBC.0b013e3283333c7c

[5] Andrew M Paes B Milner R Development of the human coagulation system in the healthy premature infant Blood 1988 72 05 1651 1657 3179444

[6] Bhat R Kumar R Kwon S Murthy K Liem R I Risk factors for neonatal venous and arterial thromboembolism in the neonatal intensive care unit-a case control study J Pediatr 2018 195 28 32 29398052 10.1016/j.jpeds.2017.12.015

[7] Bhatt M D Chan A K Venous thrombosis in neonates Fac Rev 2021 10 20 33718937 10.12703/r/10-20 PMC7946391

[8] Canadian Neonatal Network Investigators El-Naggar W Yoon E W McMillan D Epidemiology of thrombosis in Canadian neonatal intensive care units J Perinatol 2020 40 07 1083 1090 32385393 10.1038/s41372-020-0678-1

[9] Schmidt B Andrew M Neonatal thrombosis: report of a prospective Canadian and international registry Pediatrics 1995 96(5 Pt 1):939 943 7478839

[10] Sirachainan N Limrungsikul A Chuansumrit A Incidences, risk factors and outcomes of neonatal thromboembolism J Matern Fetal Neonatal Med 2018 31 03 347 351 28110589 10.1080/14767058.2017.1285892

[11] Unal S Gönülal D Siyah Bilgin B Koşan Çulha V Yarali N Experience and prognosis of systemic neonatal thrombosis at a level III NICU J Pediatr Hematol Oncol 2018 40 07 e410 e414 29750744 10.1097/MPH.0000000000001218

[12] Monagle P Adams M Mahoney M Outcome of pediatric thromboembolic disease: A report from the Canadian Childhood Thrombophilia Registry Pediatr Res 2000 47 06 763 766 10832734 10.1203/00006450-200006000-00013

[13] Yang L Ye L Lin R The use of alteplase in a newborn with an aortic arch thrombus during extracorporeal membrane oxygenation Cardiol Young 2019 29 10 1307 1309 31475664 10.1017/S1047951119002026

[14] Weiner G M Castle V P DiPietro M A Faix R G Successful treatment of neonatal arterial thromboses with recombinant tissue plasminogen activator J Pediatr 1998 133 01 133 136 9672526 10.1016/s0022-3476(98)70192-1

[15] Ferrari F Vagnarelli F Gargano G Early intracardiac thrombosis in preterm infants and thrombolysis with recombinant tissue type plasminogen activator Arch Dis Child Fetal Neonatal Ed 2001 85 01 F66 F69 11420328 10.1136/fn.85.1.F66 PMC1721288

[16] For the Childhood Thrombophilia Study Group Heller C Schobess R Kurnik K Abdominal venous thrombosis in neonates and infants: Role of prothrombotic risk factors - a multicentre case-control study Br J Haematol 2000 111 02 534 539 11122096 10.1046/j.1365-2141.2000.02349.x

[17] Kim J H Lee Y S Kim S H Lee S K Lim M K Kim H S Does umbilical vein catheterization lead to portal venous thrombosis? Prospective US evaluation in 100 neonates Radiology 2001 219 03 645 650 11376248 10.1148/radiology.219.3.r01jn17645

[18] Morag I Epelman M Daneman A Portal vein thrombosis in the neonate: risk factors, course, and outcome J Pediatr 2006 148 06 735 739 16769378 10.1016/j.jpeds.2006.01.051

[19] Hartmann J Hussein A Trowitzsch E Becker J Hennecke K H Treatment of neonatal thrombus formation with recombinant tissue plasminogen activator: Six years experience and review of the literature Arch Dis Child Fetal Neonatal Ed 2001 85 01 F18 F22 11420316 10.1136/fn.85.1.F18 PMC1721267

[20] Thrombosis, Hemostasis in Newborns (THiN) Group Leong R Patel J Samji N Use of thrombolytic agents to treat neonatal thrombosis in clinical practice Blood Coagul Fibrinolysis 2022 33 04 193 200 35285449 10.1097/MBC.0000000000001134

[21] Anderson B Urs P Tudehope D Ward C The use of recombinant tissue plasminogen activator in the management of infective intracardiac thrombi in pre-term infants with thrombocytopaenia J Paediatr Child Health 2009 45 10 598 601 19825023 10.1111/j.1440-1754.2009.01572.x

[22] Erdinç K Sarıcı S U Dabak O A neonatal thrombosis patient treated successfully with recombinant tissue plasminogen activator Turk J Haematol 2013 30 03 325 327 24385815 10.4274/Tjh.07641 PMC3878541

[23] Kayıran P G Gürakan B Kayıran S M Successful treatment of arterial thrombus in an extremely low-birth-weight preterm neonate Pediatr Neonatol 2013 54 01 60 62 23445745 10.1016/j.pedneo.2012.10.006

[24] Kimura S Takahashi K Kaneyasu H Successful treatment of saddle pulmonary thromboembolism in 23-week preterm infant Int Heart J 2022 63 05 989 994 36104227 10.1536/ihj.21-838

[25] Kurimoto T Shimoji Y Shimabukuro A Ohshiro T Recombinant tissue-type plasminogen activator treatment in an extremely low birth weight infant Clin Case Rep 2021 9 05 e04236 34026194 10.1002/ccr3.4236 PMC8123542

[26] Manco-Johnson M J Nuss R Hays T Krupski W Drose J Manco-Johnson M L Combined thrombolytic and anticoagulant therapy for venous thrombosis in children J Pediatr 2000 136 04 446 453 10753241 10.1016/s0022-3476(00)90006-4

[27] Grizante-Lopes P Garanito M P Celeste D M Krebs V LJ Carneiro J DA Thrombolytic therapy in preterm infants: Fifteen-year experience Pediatr Blood Cancer 2020 67 10 e28544 32710708 10.1002/pbc.28544

[28] Gupta A A Leaker M Andrew M Safety and outcomes of thrombolysis with tissue plasminogen activator for treatment of intravascular thrombosis in children J Pediatr 2001 139 05 682 688 11713447 10.1067/mpd.2001.118428

[29] Pediatric Coagulation Consortium Wang M Hays T Balasa V Low-dose tissue plasminogen activator thrombolysis in children J Pediatr Hematol Oncol 2003 25 05 379 386 12759624 10.1097/00043426-200305000-00006

[30] Holden R W Plasminogen activators: Pharmacology and therapy Radiology 1990 174(3 Pt 2):993 1001 2137641 10.1148/radiology.174.3.174-3-993

[31] Michelson A D Bovill E Andrew M Antithrombotic therapy in children Chest 1995 108(4 Suppl):506S 522S 7555200 10.1378/chest.108.4_supplement.506s

[32] Childhood Thrombophilia Study Group Nowak-Göttl U Junker R Kreuz W Risk of recurrent venous thrombosis in children with combined prothrombotic risk factors Blood 2001 97 04 858 862 11159508 10.1182/blood.v97.4.858

[33] Andrew M Vegh P Johnston M Bowker J Ofosu F Mitchell L Maturation of the hemostatic system during childhood Blood 1992 80 08 1998 2005 1391957

[34] Raffini L Thrombolysis for intravascular thrombosis in neonates and children Curr Opin Pediatr 2009 21 01 9 14 19242237 10.1097/MOP.0b013e32831ef537

[35] Haemostasis and Thrombosis Task Force, British Committee for Standards in Haematology Williams M D Chalmers E A Gibson B E The investigation and management of neonatal haemostasis and thrombosis Br J Haematol 2002 119 02 295 309 12406062 10.1046/j.1365-2141.2002.03674.x

[36] Will A Neonatal haemostasis and the management of neonatal thrombosis Br J Haematol 2015 169 03 324 332 25597831 10.1111/bjh.13301

[37] Manco-Johnson M J How I treat venous thrombosis in children Blood 2006 107 01 21 29 16099882 10.1182/blood-2004-11-4211 PMC1895344

[38] Newall F Barnes C Ignjatovic V Monagle P Heparin-induced thrombocytopenia in children J Paediatr Child Health 2003 39 04 289 292 12755937 10.1046/j.1440-1754.2003.00139.x

[39] Andrew M Marzinotto V Massicotte P Heparin therapy in pediatric patients: a prospective cohort study Pediatr Res 1994 35 01 78 83 8134203 10.1203/00006450-199401000-00016

[40] Monagle P Chan A KC Goldenberg N A Antithrombotic therapy in neonates and children: Antithrombotic Therapy and Prevention of Thrombosis, 9th ed: American College of Chest Physicians Evidence-Based Clinical Practice Guidelines Chest 2012 141(2 Suppl):e737S e801S 22315277 10.1378/chest.11-2308 PMC3278066

[41] Newall F Ignjatovic V Summerhayes R In vivo age dependency of unfractionated heparin in infants and children Thromb Res 2009 123 05 710 714 18829072 10.1016/j.thromres.2008.07.009

